# Supplementary material for: Zebrafish CCNF and FUS Mediate Stress-Specific Motor Responses
Source: Cells. 2024 Feb 21;13(5):372. doi: 10.3390/cells13050372 (PMC10930717; doi:10.3390/cells13050372)
Supplement: Supplementary file 1 [file cells-13-00372-s001.zip › cells-2822235-supplementary.pdf]

Supplementary Information

Supplementary Table S1. Primers used for sgRNA production and genotyping.

Oligo #	Oligo Name	5' - Sequence - 3'	GC%	Length	Tm
MND111	CCNF-SGEK500-F	CATTATTAATTCAAGGGGGCGCTAT	40	25-mer	70C
MND112	CCNF-SGEK500-R	CCACAACTGCTTACTCTGAAACA	43	23-mer	66C
MND113	CCNF-SGEK100-F	CAGAGTTGTCCAATCAGGAGGAGA	50	24-mer	72C
MND114	CCNF-SGEK100-R	AAACACGTCAATATCCCGCTAACA	42	24-mer	68C
MND120	CCNF-E624K-F	TCCCTCgctCTCACGGTCT	53	19-mer	52C
MND124	CCNF_ex16_HRM1_R	TATGATTTGAGCCGTTTCATGC	41	22-mer	62C
MND129	CCNF_int16_HRM1_F	TGGATTCTACCACTGACTCTGATG	46	24-mer	70C
MND130	CCNF_int16_HRM1_R	GCCCCTATATTTTCCACAACTGC	48	23-mer	68C
MND133	CCNF_ex16_HRM2_F	CCTGCTGGGAGACTTTCTGG	60	20-mer	64C
MND134	CCNF_ex16_HRM2_R	GCAGGTTCTGAAACACTGCT	50	20-mer	60C
MND-C01	sgRNA_ex14_L1_F	TAGGGGGCACATCTTGACCAAAAC	50	24-mer	72C
MND-C02	sgRNA_ex14_L1_R	AAACGTTTTGGTCAAGATGTGCCC	46	24-mer	70C
MND-C03	sgRNA_ex14_L2_F	TAGGGACACGTGTCTGTAGTCTTT	46	24-mer	70C
MND-C04	sgRNA_ex14_L2_R	AAACAAAGACTACAGACACGTGTC	42	24-mer	68C
MND-C05	sgRNA_ex14_U1_F	TAGGTACAGACACGTGTCATTAAC	42	24-mer	68C
MND-C06	sgRNA_ex14_U1_R	AAACGTTAATGACACGTGTCTGTA	38	24-mer	66C
MND-C07	sgRNA_ex14_U2_F	TAGGCATTAACTGGAGTCAAGCAA	42	24-mer	68C
MND-C08	sgRNA_ex14_U2_R	AAACTTGCTTGACTCCAGTTAATG	38	24-mer	66C
MND-C09	sgRNA_ex16_L1_F	TAGGATCCAGAACAGGAAGTGTCC	50	24-mer	72C
MND-C10	sgRNA_ex16_L1_R	AAACGGACACTTCCTGTTCTGGAT	46	24-mer	70C
MND-C11	sgRNA_ex16_L2_F	TAGGGTCTCCCTCGTATCCAGAAC	54	24-mer	74C
MND-C12	sgRNA_ex16_L2_R	AAACGTTCTGGATACGAGGGAGAC	50	24-mer	72C
MND-C13	sgRNA_ex16_L3_F	TAGGCCTTCTCTCCCTCGCTCTCA	58	24-mer	76C
MND-C14	sgRNA_ex16_L3_R	AAACTGAGAGCGAGGGAGAGAAGG	54	24-mer	74C
MND-C15	sgRNA_ex16_U1_F	TAGGCCGTGAGAGCGAGGGAGAGA	63	24-mer	78C
MND-C16	sgRNA_ex16_U1_R	AAACTCTCTCCCTCGCTCTCACGG	58	24-mer	76C

Supplementary Table S2. Cutting efficiency of eight sgRNAs targeting *ccnf* was determined using RFLP.

ID	Binding Site	Target	Cutting Efficiency (%)	RFLP Enzyme
sgRNA_ex14_L1	GGGCACATCTTGACCAAAAC	*ccnf*	0%	**BsrI**
sgRNA_ex14_L2	GACACGTGTCTGTAGTCTTT	*ccnf*	0%	**SfcI**
sgRNA_ex14_U1	TACAGACACGTGTCATTAAC	*ccnf*	16%	**AflIII**
sgRNA_ex14_U2	CATTAACTGGAGTCAAGCAA	*ccnf*	5%	**PleI**
sgRNA_ex16_L1	ATCCAGAACAGGAAGTGTCC	*ccnf*	0%	**BstNI**
sgRNA_ex16_L2	GTCTCCCTCGTATCCAGAAC	*ccnf*	0%	**BciVI**
sgRNA_ex16_L3	CCTTCTCTCCCTCGCTCTCA	*ccnf*	64%	**HpyCH4III**
sgRNA_ex16_U1	CCGTGAGAGCGAGGGAGAGA	*ccnf*	45%	**HpyAV**

Supplementary Table S3. TALEN designs targeting zebrafish *fus* gene.

TALE #	Plus strand sequence	Unique restriction sites in spacer	% RVDs HD or NN/NH
**Tale01**	T GTTACAGTGCAGGAGCA ggataactctgacaa CAACACCATCTTTGTAC A	Hpy188I	47
**Tale02**	T GACAACAACACCATCTT tgtacaaggtcttgg AGACGACTATACTGTGG A	CviQI	44
**Tale03**	T GACTCATGGCTTCAAAA agttctgcgctaaatg TCTAGTTGTTTTCTCAC A	HinP1I	38
**Tale04**	T GGCTTCAAAAAGTTCTG cgctaaatgtctagtt GTTTTCTCACACAGGTG A	BfaI	44
**Tale05**	T CTAGTTGTTTTCTCACA caggtgaataagaaga CAGGATTGCCCATGATT A	HphI	41
**Tale06**	T TTTCTCACACAGGTGAA taagaagacaggatt GCCCATGATTAATCTGT A	BbsI	41
**Tale07**	T GAATAAGAAGACAGGAT tgcccatgattaatc TGTATACAGACAGAGAG A	AseI	38

**Tale08**	T ACAGACAGAGAGACGGG gaagcttaaagggga GGCTACTGTTTCCTTTG A	HindIII	53
**Tale09**	T ACTGTGGACTCTGTTGC agactactttaaacag ATCGGCATCATCAAGGT A	None	50
**Tale10**	T GATTAATCTGTATACAG acagagagacgggga AGCTTAAAGGGGAGGCT A	None	41
**Tale11**	T CAGCAAAAGCAGCCATT gattggtttgatggt ACGTTGAAGAGGATGAC A	BccI	47

Supplementary Table S4. Primers used for TALEN production and genotyping.

Oligo #	Oligo Name	5' - Sequence - 3'	GC%	Length	Tm
**MND215**	zFUS_HRM_Out1_F	GCAGGAGCAGGATAACTCTGA	52	21-mer	64C
**MND216**	zFUS_HRM_Out1_R	TGCCTTGCCACTTTACCTTGA	48	21-mer	62C
**MND217**	zFUS_HRM_In1_F	GGACTCTGTTGCAGACTACTTTAAA	40	25-mer	70C
**MND218**	zFUS_HRM_Out2_F	AGAAGACAGGATTGCCCATGA	48	21-mer	62C
**MND219**	zFUS_HRM_Out2_R	TGCTGAAGGAGGGTCATCAA	50	20-mer	60C
**MND220**	zFUS_HRM_In2_F	ACAGACAGAGAGACGGGGAA	55	20-mer	62C
**MND286**	zFUS_KO_Seq1_F	CTGCGTTGCTCTGTACATGC	55	20-mer	62C
**MND287**	zFUS_KO_Seq1_R	CCACGACCAAACTCAGCTCT	55	20-mer	62C
**MND288**	zFUS_KO_Seq2_F	TGGTGGGCCCAATAGTAAGTCAAA	46	24-mer	70C
**MND289**	zFUS_KO_Seq2_R	GTGTTGTGAGGGGTTTTACAGCAT	46	24-mer	70C

Supplementary Table S5. F1 progeny germline transmission rates.

Group	Screened (#)	+ve (#)	+ve (%)	%inframe (%)	%frameshift (%)
CCNF	86	11	13%	64%	36%

Line #	5' - Sequence – 3	Indel
0	5' GACCGTGAG--------------AGCGAGGGAGAGAAGG 3'	WT
1	5' GACCG---G--------------AGCGAGGGAGAGAAGG 3'	-3
2	5' GACCGTGAGAC------------AGCGAGGGAGAGAAGG 3'	+2
3	5' GACCGTGAG--------------------GGAGAGAAGG 3'	-6
4	5' GACCGTGAG--------------AGC----GAGAGAAGG 3'	-4
5	5' GACCGTGAGGGG-----------AGCGAGGGAGAGAAGG 3'	+3
6	5' -------AG--------------AGCGAGGGAGAGAAGG 3'	-7
7	5' GACCGTGAGGCA-----------AGCGAGGGAGAGAAGG 3'	+3
8	5' GACCGTGAGGGTACGCCA-----AGCGAGGGAGAGAAGG 3'	+9
9	5' GACCGT---------------AGCGAGGGAGAGAAGG 3'	-3
10	5' GACCGTGAG-----------------------GAGAAGG 3'	-9
11	5' GACCGTGAGGGACCGTGAGGGAGAGCGAGGGAGAGAAGG 3'	+14

Supplementary Table S6. F1 progeny germline transmission rates.

Group	Screened (#)	+ve (#)	+ve (%)	%inframe (%)	%frameshift (%)
FUS	38	9	24%	78%	22%

Line #	5' - Sequence – 3	Indel
0	GGACTCTGTTGCAGAC------TACTTTAAACAGATCGGCATCATC	WT
1	GGACTCTGTTGCAGAC----------------AGATCGGCATCATC	-10
2	GGACTCTGTTGCAGACCAGCCGTACTTTAAACAGATCGGCATCATC	+6
3	GGACTCTGTTGCAGAC------CACTTTAAACAGATCGGCATCATC	+/-1
4	GGACTCTGTTGCAGAC---------TTTAAACAGATCGGCATCATC	-3
5	GGACTCTGTTGCAGAC-------------------TCGGCATCATC	-13
6	GGACTCTGTTGCAGACGGT---TACTTTAAACAGATCGGCATCATC	+3
7	GGACTCTGTTGCAGACGTGAGGTACTTTAAACAGATCGGCATCATC	+6
8	GGACTCTGTT------------TACTTTAAACAGATCGGCATCATC	-6
9	GGACTCTGTTGCAGAC---------------CAGATCGGCATCATC	-9

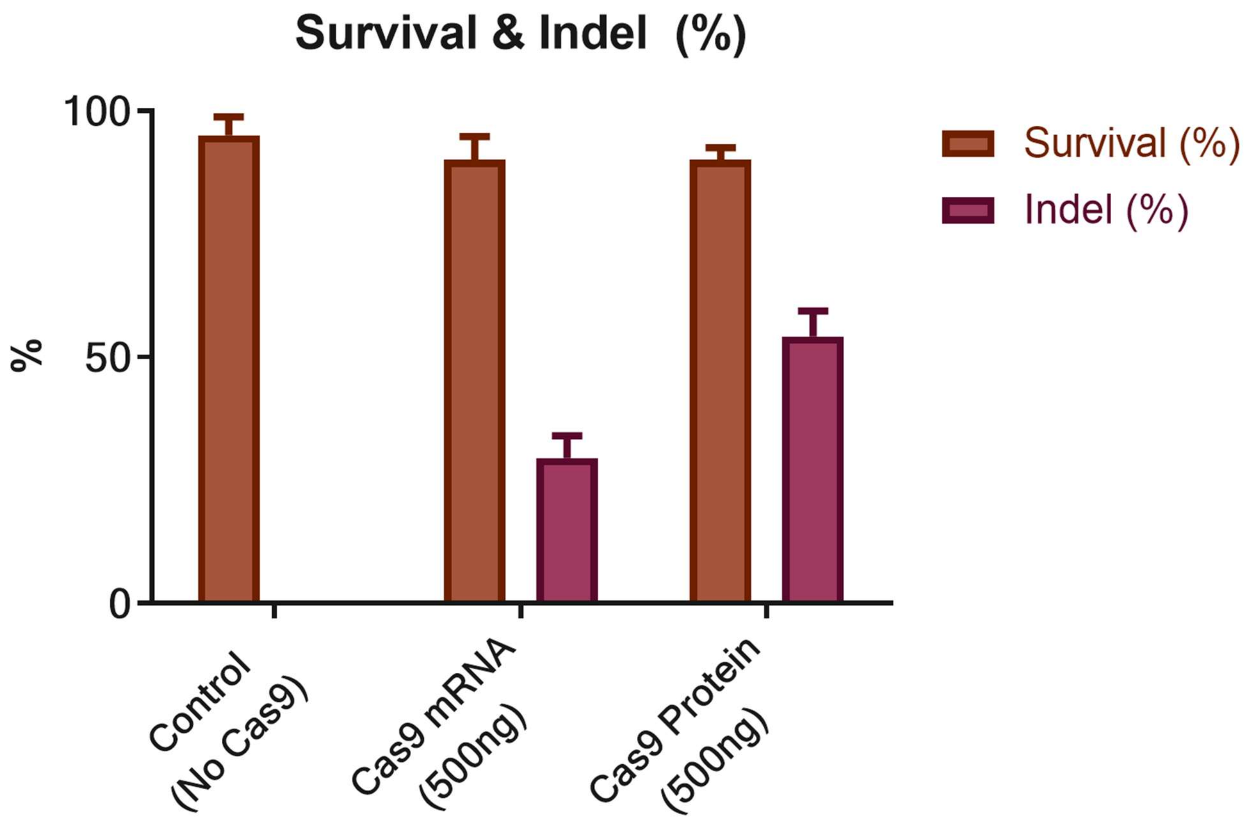

Supplementary Figure S1. Effect of Cas9 mRNA and Cas9 Protein in zebrafish embryo survival and F0 indel rate (%).

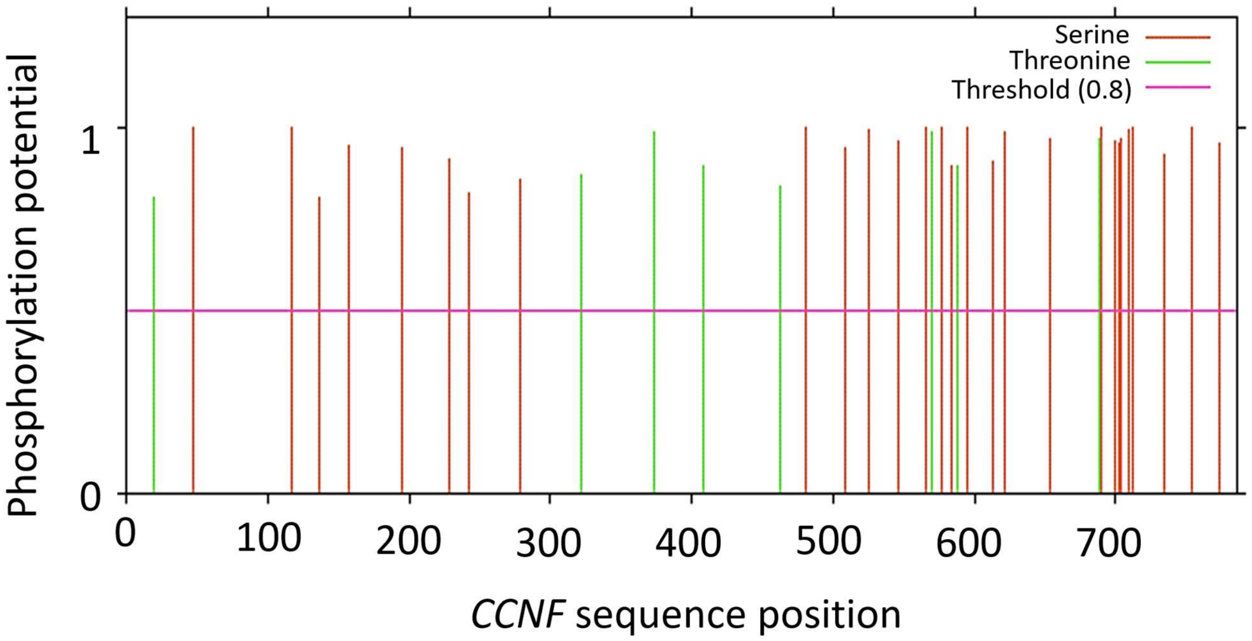

Supplementary Figure S2. Predicted phosphorylation sites of CCNF (Netphos 3.1a).

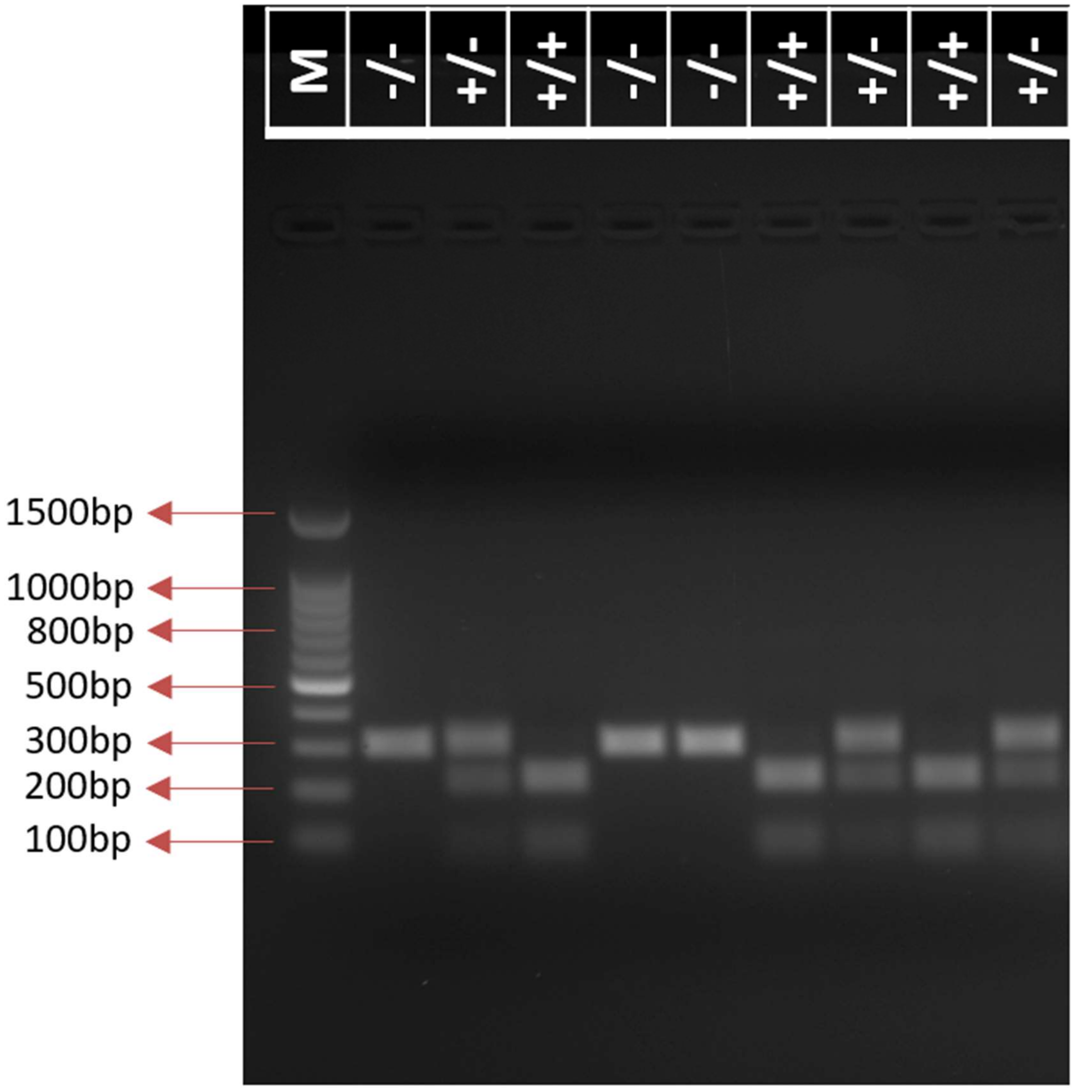

Supplementary Figure S3. Rapid genotyping using RFLP. F1 progeny carrying endogenous wild-type *fus* gene contains unique DraI restriction site in the PCR product amplified using primers 215/219, whereas homogenous *fus* mutants (fus$^{-/-}$) lack the restriction site.

A

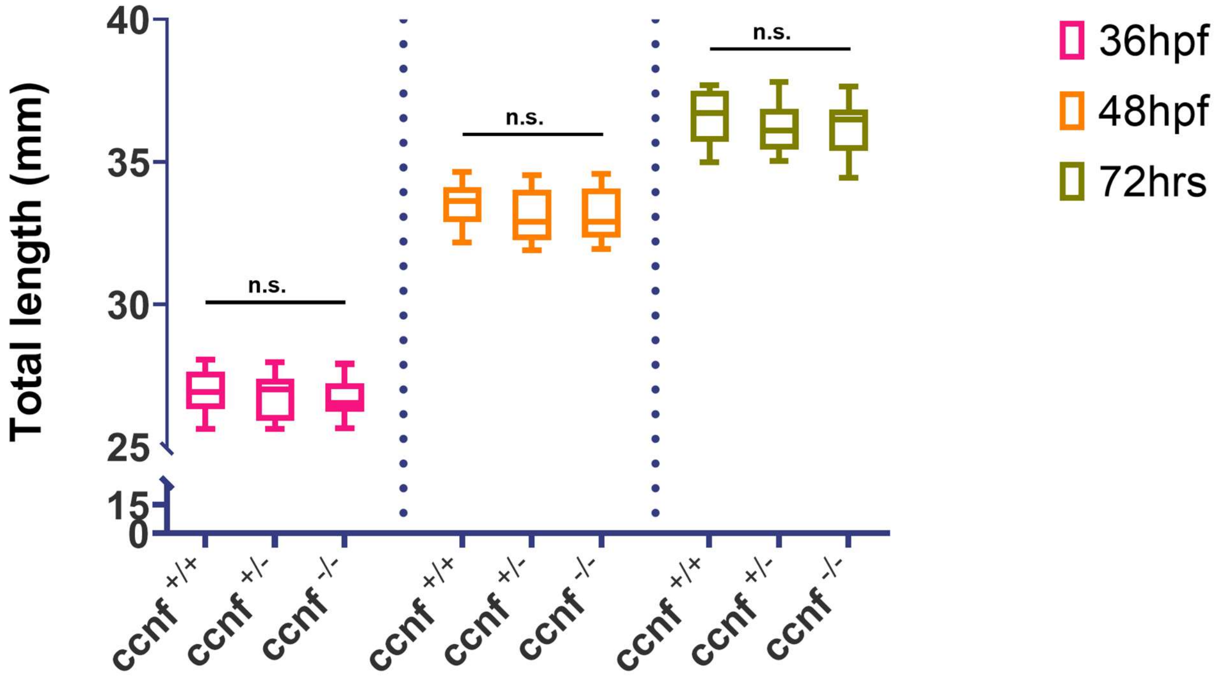
Total length (mm)
40
35
30
25
15
0
n.s.
n.s.
n.s.
ccnf +/+
ccnf +/-
ccnf -/-
ccnf +/+
ccnf +/-
ccnf -/-
ccnf +/+
ccnf +/-
ccnf -/-
36hpf
48hpf
72hrs

B

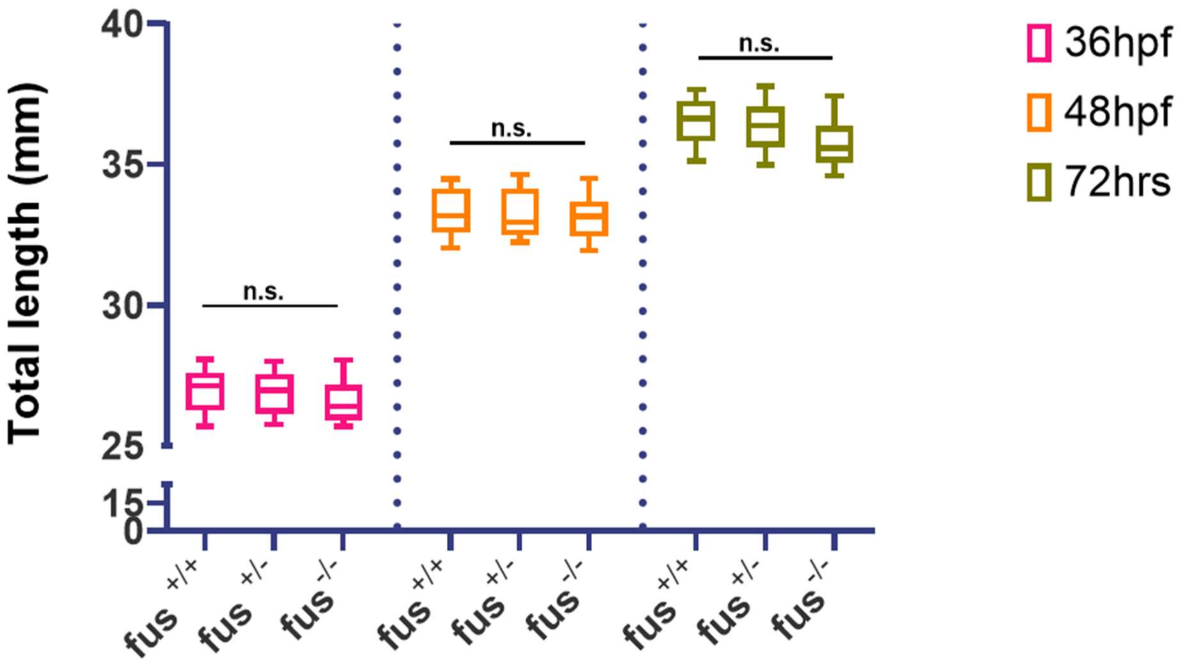
Total length (mm)
40
35
30
25
15
0
n.s.
n.s.
n.s.
fus +/+
fus +/-
fus -/-
fus +/+
fus +/-
fus -/-
fus +/+
fus +/-
fus -/-
36hpf
48hpf
72hrs

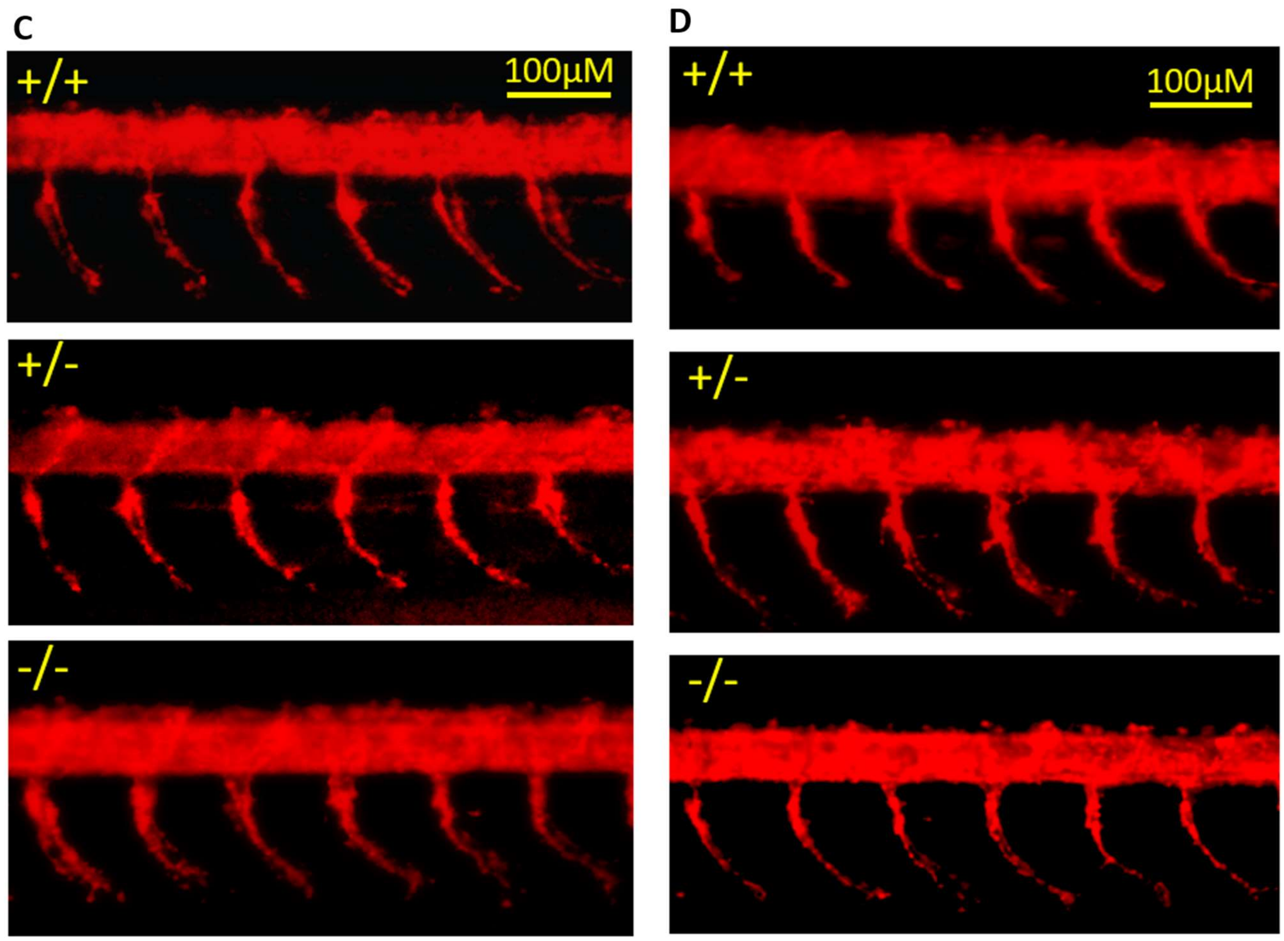

Supplementary Figure S4. The total length of zebrafish larvae at multiple time points.

(A,B) The average length of six motor neurons above the yolk sac extension were analysed in all three genotypes of ccnf (n=41 fish) and fus (n=90). Representative images SV2 stained motor neurons of CCNF (C) and FUS (D) zebrafish larvae at 48hpf.

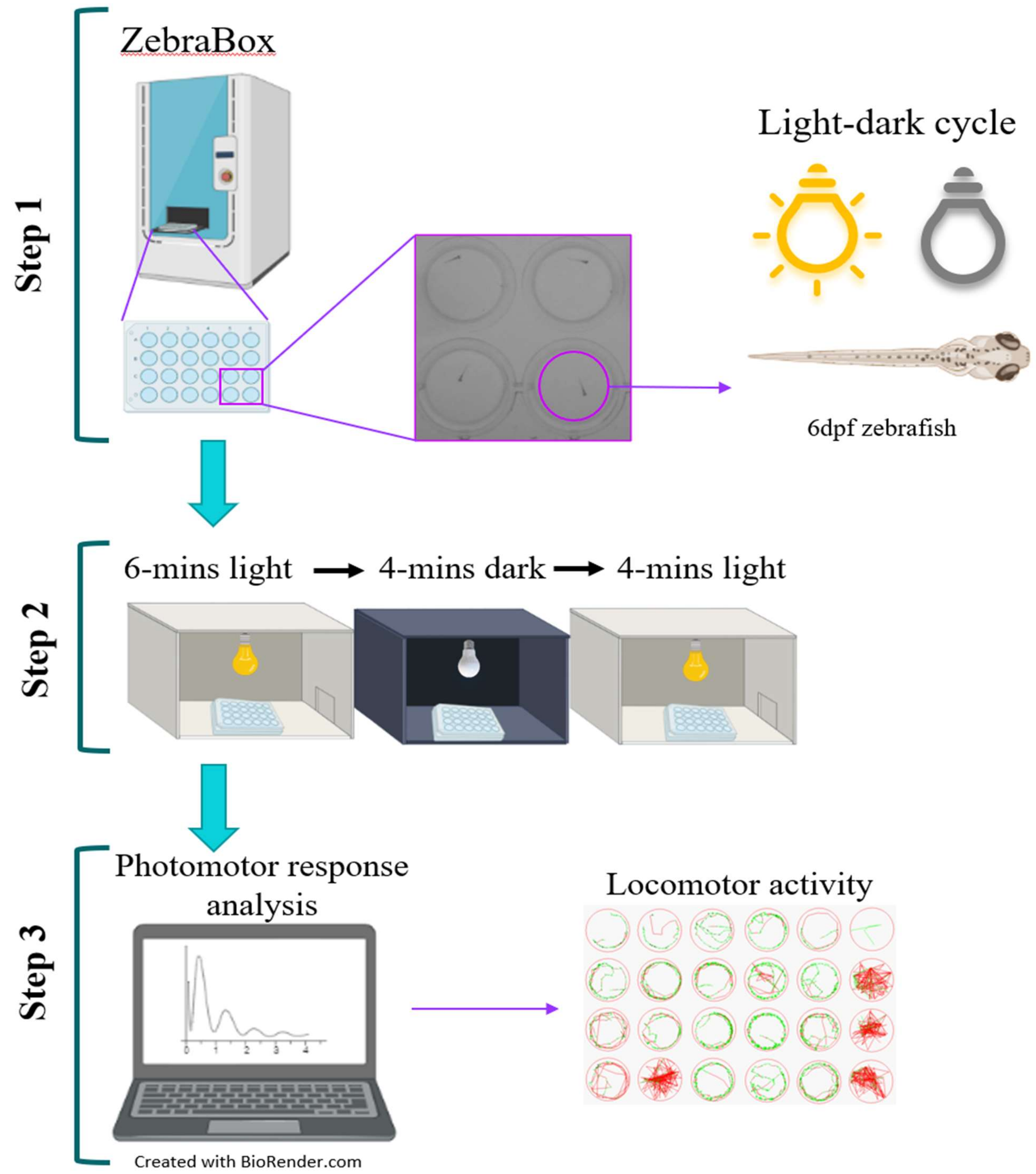

Supplementary Figure S5. Photomotor response and locomotor activity. Zebrafish larvae (6dpf) are plated in a 24-well plate, then placed into Viewpoint Zebrabox (Step 1). Zebrafish larvae are subjected to light-dark-light cycle (Step 2). The transition from light to dark increases zebrafish locomotor activity. Zebrafish larvae photomotor response and locomotor activity are recorded by ZebraLab software. Photomotor response analysis (total distance travelled and average velocity) is conducted (Step 3).

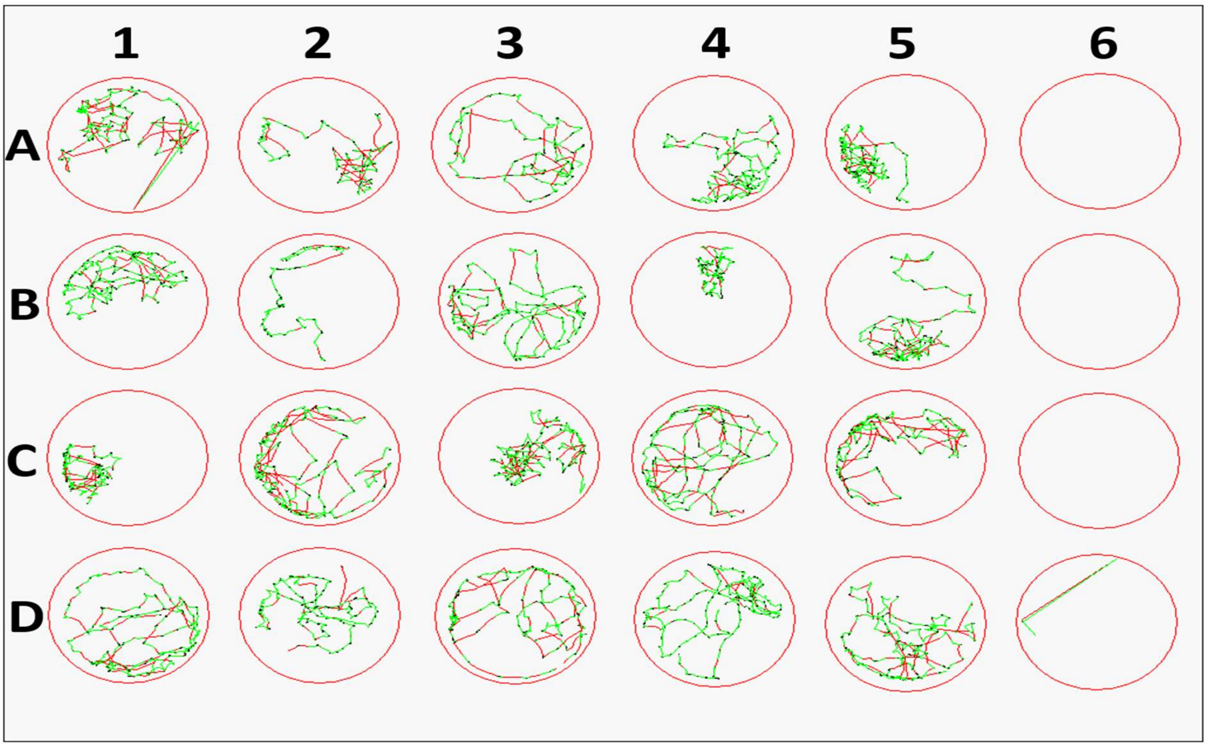

	1	2	3	4	5	6
A	WT	Het	Het	WT	Het	
B	Het	Homo	WT	Homo	Het	
C	Homo	WT	Het	Het	WT	
D	Het	Het	WT	Het	Het	

Supplementary Figure S6. Representative swimming trajectories of zebrafish embryos (*ccnf* mutants and wild-type controls) at 6dpf.

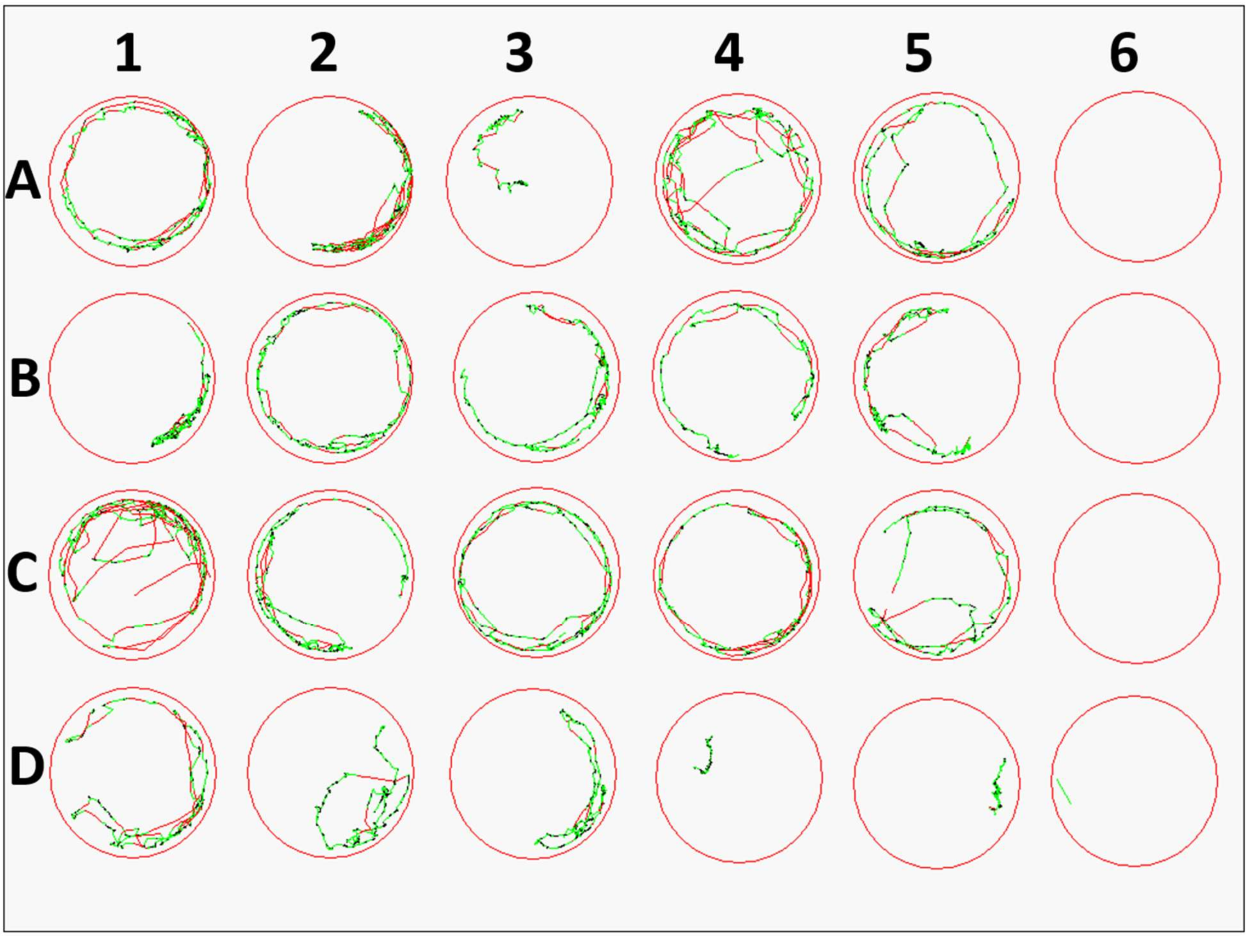

P2	1	2	3	4	5	6
A	het	het	homo	WT	het	
B	het	WT	het	WT	het	
C	het	het	het	WT	WT	
D	het	het	het	homo	homo	

Supplementary Figure S7. Representative swimming trajectories of zebrafish embryos (*fus* mutants and wild-type controls) at 6dpf.

Supplementary Information

Supplementary Methods S1. Design and production of TALENs targeting zebrafish *fus* gene**.** **Samples:**

#	TALEs	ID#	TALE RVDs
1	zFUS _KO_01	Tale09F	NI HD NG NN NG NN NN NI HD NG HD NG NN NG NG NN HD
		Tale09R	NI HD HD NG NG NN NI NG NN NI NG NN HD HD NN NI NG
2	zFUS _KO_02	Tale10F	NN NI NG NG NI NI NG HD NG NN NG NI NG NI HD NI NN
		Tale10R	NI NN HD HD NG HD HD HD HD NG NG NG NI NI NN HD NG

TALEN R#1

1. Label 2sets of (2x8) 0.2ml PCR strip tubes:
    - TAL_1_a3a, TAL_1_a3b, TAL_1_a2b, TAL_1_b2
    - TAL_2_a3a, TAL_2_a3b, TAL_2_a2b, TAL_2_b2
2. Thaw, spin, vortex and spin the module plasmid library of RVD samples
3. Add 0.9µl of the respective TALE RVDs to each tube
4. Add 0.9µl of the respective TALE Destination Vector to each
5. Add 1.5µl of TALE R#1 Master Mix to each tube
6. Run TALE R#1 program:
7. Additional Digestion: add 1.3µl of the Add-Dig-Mastermix
8. Run TALE R#1 program again

TALE R#1 Master Mix		
**Component**	**1x (µl)**	**18x (µl)**
T4-Ligase Buffer (10X)	0.6	10.8
T4 DNA Ligase Enzyme	0.3	5.4
BsaI Enzyme	0.3	5.4
ATP (25 mM)	0.24	4.3
Ultra Pure H_2O	0.06	1.1
**Total**	**1.5**	**27.0**

TALE R#1 Program		
Temp	Time	Rep
37°C	5’	X3
16°C	10’	
4°C	**2’**	x1

Transformation

9. Set water bath to 42°C
10. Label 16 x 1.5ml Eppendorf tubes
11. Thaw competent *E.coli* cells on ice (NEB 10-beta stored on -80°C)
12. Add 10µl of thawed *E.coli* cells to each tube
13. Add 1µl of ligations to each tube
14. Mix gently by flicking the tubes then incubate on ice for 20mins
15. Heat-Shock the cells at 42°C for 30s (Do Not Shake)

Add-Dig Master Mix		
**Component**	**1x (µl)**	**18x (µl)**
CutSmart Buffer (10X)	1.0	18.0
BsaI Enzyme	0.3	5.4
**Total**	**1.3**	**23.4**

16. Immediately return the tubes to ice and incubate for 2mins
17. Add 500µl of SOC-medium (stored at 4°C) then incubate at 37°C for 1hr
18. Plate transformation culture onto LB-Spec-Xgal+IPTG plates. From each transformation, plate 30µl on to one plate, and the rest (480µl) on to another plate.
19. Incubate plates upside down overnight at 37°C

Colony PCR

20. Label 0.2ml PCR strip tubes
21. From each plate, pick a single white colony with a pipette and mix in 20µl Ultrapure H_2O. Do this twice for each construct.
22. Pick one single blue colony as negative control and mix in 20µl Ultrapure H_2O
23. Split the mixtures into two – keep one in the fridge for liquid culture
24. Heat 10µl samples at 99°C for 10mins.
25. Spin the samples for 3mins to pellet cell debris
26. Prepare Colony PCR Mastermix
27. Add 7µl from Colony PCR Mastermix to 0.2ml tubes
28. Add 3µl from the template DNA in each tube
29. Run PCR using the relevant Colony PCR Program
30. Run PCR products on 2% Agarose gel with a 100bp ladder. Expect bands as shown below. Plasmid b2 contains only 2 RVDs, so the fragment size is smaller than the other intermediate array plasmids, which contain 4 RVDs. The negative control (unsuccessful ligation) should show slightly larger fragment size than the correctly ligated plasmids.

**Colony PCR Mastermix**			
**Component**	**1x (µl)**	**36x (µl)**	**Final**
Ultrapure H_2O	1.5	54.0	
GoTaq Green	5.0	230.0	1X
Primer pair 104 & 105 10uM each	0.5	18.0	0.5 µM
Template DNA	(3.0)		
**Total**	**10.0**		

	**Colony PCR Program**		
	Temp	Time	Rep
Step-1	95°C	2’	
Step-2	95°C	15’’	
Step-3	**55°C**	15’’	x30
Step-4	72°C	**105’’**	
Step-5	72°C	10’	
Step-6	10°C	2’	

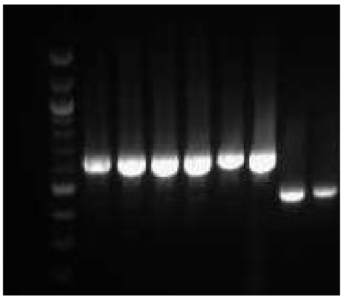

Liquid Culture of Positive Constructs

31. Inoculate the 10µl (see step 21) of positive hits from Colony PCR in 5ml 2YT medium + Spec in 15ml Falcon tubes. Take off original lids and use pink and white loose lids, to allow O_2 circulation.
32. Incubate for 16 hours at 37°C on rotator.

MiniPrep

Using BIONEER *Accuprep*© Nano-Plus Plasmid Mini Extraction Kit, short protocol

33. Harvest the 5ml of cultured E coli cells by centrifugation for 5 mins at 3000 X g (or 2 min at >8000rpm), and remove media by pouring out then pipetting.
34. Add 250µl of Buffer (1) (in fridge) to the collected pellet and resuspend by pipetting. Shake buffer well before each use. Transfer resuspension to labelled 1.5ml Eppendorf tubes.
35. Add 250µl of Buffer (2) and mix by inverting tube 3-4 times, gently.
36. Add 350µl of Buffer (3) and immediately mix by inverting 3-4 times, gently.
37. Centrifuge the tubes for 1min at 4°C, 13,000rpm.
38. Transfer the cleared lysate (800µl) to the labelled DNA binding column and centrifuge for 1min at 13,000rpm. Take care to not touch the white powder pellet when transferring the lysate.
39. Pour off the flow-through and reassemble the DNA binding tube with the 2ml collection tube.
40. Add 700µl of fresh 80% ethanol [in place of Buffer (4)] to the DNA binding column, and centrifuge for 1 min at 13,000rpm.
41. Transfer the column to labelled 1.5ml eppendorf tubes and centrifuge for 1min at 13,000rpm to dry the column.
42. Transfer the DNA binding filter column to a new labelled 1.5µl eppendorf tube, and add 100µl of 10mM Tris pH 8.0 to elute the plasmid. Wait 2 mins then centrifuge for 1 min at 13,000rpm. Discard the column and measure DNA yield with the Nanodrop machine.
43. Dilute plasmids to 50ng/µl, using 10mM Tris pH 8.0.

**TALEN R#2 Master Mix**		
**Component**	**1x (µl)**	**2.5x (µl)**
Ultrapure H_2O	3.0	7.5
T4-Ligase Buffer (10X)	0.8	2.0
T4 DNA Ligase Enzyme	0.4	1.0

Esp3I Enzyme	0.4	1.0
ATP (25mM)	0.4	1.0
Total	5	12.5

TALEN R#2

Ligation of intermediate arrays into destination vectors

44. Label 2 x 0.2ml PCR tubes: TAL_1 and TAL_2
45. Thaw, spin, vortex and spin the 50ng/μl vectors (a3a, a3b, a2b, b4)
46. Add 1.2ul of the respective vectors (a3a, a3b, a2b, b4) to each tube [refer to construction template]
47. Add 0.6ul of the respective Destination Vector (e.g. 63_NN) to each tube
48. Add 5μl of TALE R#2 Master Mix to each tube
49. Run TALE R#2 program

TALE R#2		
Temp	Time	Rep
37°C	5’	x12
16°C	10’	
37°C	**15’**	
80 °C	**5’**	
4 °C	**2’**	

Transformation

50. Set water bath to 42°C
51. Label 1.5ml Eppendorf tubes
52. Thaw competent *E.coli* cells on ice (NEB 10-beta stored on -80)
53. Add 10μl of thawed *E.coli* cells to each tube
54. Add 2μl of ligations to each tube
55. Mix gently by flicking the tubes then incubate on ice for 20mins
56. Heat-Shock the cells at 42°C for 30s (Do Not Shake)
57. Immediately return the tubes to ice and incubate for 2mins
58. Add 500μl of SOC-medium (stored at +4C) then incubate at 37°C for 1hrs
59. Plate the whole 510μl of transformation culture onto LB-Amp-Xgal+IPTG plates
60. Incubate plates upside down overnight at 37°C
61. Select 3 white colonies from each plate and inoculate into 5ml 2YT + Amp, and leave on rotator overnight
62. Follow mini-prep protocol as before

MscI Digestion

#R0534S MscI 5U/ul / MscI cuts at every NI RVD

MscI Mix		
**Component**	**1x (μl)**	**3x (μl)**
10x CutSmart Buffer	3.5	10.5
MscI Enzyme	1	3
Plasmid DNA	X	
UltraPureH2O	X	

Total	**35**	

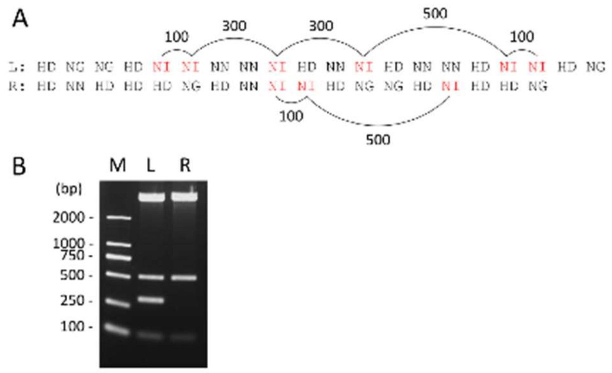

63. Label 0.2ml PCR tubes
64. 3µg Plasmid + 3.5µl 10x CutSmart Buffer + 1µl MscI + ssH_2O to fill up to a total volume of 35µl
65. digest for 2h at 37°C
66. add 7ul of 6x LoadingDye to the 35ul
67. run on 2% Agarose gel (100bp ladder)

<u>AvrII Digestion to linearise the Plasmid</u>

#R0174L AvrII 5U/µl (#R0174S AvrII 4U/µl)

**AvrII Mix**		
**Component**	**1x (µl)**	**3x (µl)**
10x CutSmart Buffer	3.5	10.5
AvrII Enzyme	1.5	4.5
Plasmid DNA	X	
ssH2O	X	
Total	**35**	

68. Label 0.2ml PCR tubes
69. 7ug Plasmid + 3.5µl 10x CutSmart Buffer + 1µl AvrII + ssH2O to fill up to a total volume of 35µl
70. Digest for 2 h at 37°C
71. Take 3ul of the reaction, add 2ul H20 + 1ul 6x LoadingDye and run for 60min of a 1% Agarose gel (1kb ladder)
72. use the rest for DNA Clean & Concentrator Kit

<u>Zymo DNA Clean and Concentrator-5 Kit</u>

73. In a 1.5 ml microcentrifuge tube, add 2 volumes of DNA Binding Buffer to each volume of DNA sample (30µl linearised plasmid : 70µl DNA Binding Buffer). Mix briefly by vortexing.
74. Transfer mixture to a provided Zymo-Spin™ Column in a Collection Tube.
75. Centrifuge for 30 seconds. Discard the flow-through.
76. Add 200 µl fresh 80% ethanol to the column. Centrifuge for 30 seconds. Repeat the wash step.
77. Transfer the Zymo-Spin™ Column to a new 1.5ml microcentrifuge tube, and spin to dry the column. Repeat the spin step in a new microcentrifuge tube.

78. Add 6 µl 10mM Tris pH 8.0 directly to the column matrix and incubate at room temperature for 2 minutes. Transfer the column to a 1.5 ml microcentrifuge tube and centrifuge for 30s to elute the DNA.
79. Ultra-pure DNA is now ready for use. Nanodrop the sample to determine purity and concentration.

RNA Synthesis – T7 mMESSAGE mMACHINE® kit

80. Thaw 2 X buffer and 10 X buffer at room temperature
81. Once thawed vortex until resuspended
82. Place 10 X buffer on ice KEEP 2X buffer at room temp
83. Set up reaction (add reagents in this order):
84. 3 µl Linear DNA (or 500 ng, must be >300 ng)
85. 5 µl 2X buffer
86. 1 µl 10X buffer
87. 1 µl enzyme mix (keep enzyme on ice-block)
88. Incubate at 37C for 2 hours (or O/N)
89. Add 0.5 µl TURBO DNase (supplied with kit)
90. Incubate at 37C for 20 minutes

RNA Clean&Concentrator kit form ZYMO (R1017)

91. Follow the protocol provided with the kit
92. Elute in 50µl ultrapure H_2O
